# A Reflection on Current Definitions of Critical Care and Critical Illness—A Narrative Review of the Literature

**DOI:** 10.1111/nicc.70311

**Published:** 2026-01-08

**Authors:** Fritz Sterr, Anja Gerlach, Chris Creemers, Severin Pietsch, Julia Berkemeier, Ismail Özlü, Christina Papacek‐Zimmermann, Lydia Bauernfeind, The Division for Critical Care Nursing, German Society of Nursing Science

**Affiliations:** ^1^ Division for Critical Care Nursing German Society of Nursing Science Duisburg Germany; ^2^ Deggendorf Institute of Technology Faculty of Applied Healthcare Sciences Deggendorf Germany; ^3^ Witten/Herdecke University Faculty of Health, School of Nursing Science Witten Germany; ^4^ Department of Intensive Care Medicine University Medical Center Hamburg‐Eppendorf Hamburg Germany; ^5^ Hochschule Bielefeld ‐ University of Applied Sciences and Arts Bielefeld Faculty of Health Bielefeld Germany; ^6^ University of Cologne, Faculty of Medicine and University Hospital Cologne Institute of Nursing Science Cologne Germany; ^7^ Stiftung St. Marien Hospital Lünen Weiterbildungsstätte für Intensivpflege Und Anästhesie Sowie Notfallpflege (DKG) Lünen Germany; ^8^ University Hospital Regensburg Department for Internal Medicine II Regensburg Germany; ^9^ Paracelsus Medical University Salzburg Institute of Nursing Science and Practice Salzburg Austria

**Keywords:** chronic critical illness, critical care, critical illness, experience, intensive care unit, review

## Abstract

**Background:**

Critical care and critical illness are key concepts that have been defined in various publications. A common understanding of the concepts is of great importance, but an up‐to‐date overview and discussion are lacking in the literature.

**Aim:**

The aim of this study was to identify and discuss current definitions of critical care, critical illness and chronic critical illness.

**Study Design:**

We conducted a narrative review and performed literature searches in Medline, Cochrane Library, CINAHL, Embase, LIVIVO, Google Scholar, OpenGrey, Epistemonikos and Science Open as well as citation searching from July 2024 to September 2025. Reports on definitions were included if they dealt thematically with critical care, critical illness or chronic critical illness, were published within the last 10 years and were available in German or English. Data synthesis followed an inductive approach. We thematically analysed the core concepts of identified definitions, formed thematic clusters and finally approved them in multiple group discussions.

**Results:**

In total, 13 definitions of critical care, eight definitions of critical illness and 12 definitions of chronic critical illness were identified. Key components of critical care are the population, interventions, timing, professionals, aim, location and complexity. Critical illness is mainly characterised by future aspects and prevention, underlying causes and treatment. Chronic critical illness is determined by its duration, several complications and frailty. All three terms share the temporality and severity of a disease. Included definitions have a pronounced pathophysiological focus; they point to highly complex and technologised treatment pathways.

**Conclusion:**

Current definitions show a generally homogeneous understanding of the care for critically ill patients. To ensure a comprehensive representation, definitions need to be adapted and consider the experiences of those involved.

**Relevance to Clinical Practice:**

The analysed clusters of the definitions of critical care, critical illness and chronic critical illness help professionals to develop their terminology and reflect on their clinical approaches.

## Impact Statement

**What is known about this topic?:**

- Internationally, there are various definitions of critical care and critical illness.
- There is now a large body of literature and empirical research on these terms.

**What this paper adds?:**

- The definitions of critical care, critical illness and chronic critical illness are heterogeneous in their extent, application and evidence basis.
- Existing definitions primarily focus on urgency and timing, humans' pathophysiological deterioration and a complex treatment situation.
- The experience of patients, relatives and professionals has hardly been considered to date.

**Abbreviations**
CC: critical care
CCI: chronic critical illness
CI: critical illness
CIM: critical illness myopathy
CINM: critical illness neuromyopathy
CIP: critical illness polyneuropathy
HCP: healthcare professional
ICU: intensive care unit
LOS: length of stay

## 1 Introduction

### 1.1 Background

Worldwide, complexity in acute and critical care (CC) is continuously increasing. Next to multiple performance indicators, digitalisation, technological progress and professional role development in healthcare facilities, patients are presenting themselves with coexisting and chronic diseases, increasing age and polypharmacy [1, 2, 3, 4, 5]. In this regard, the healthcare system and its professionals have changed over the years. The demands on healthcare professionals (HCPs) are increasing, and complex care is no longer limited to a single setting [6, 7, 8]. CC in particular, which is a sub‐dimension of acute care, has become more complex and specialised in recent years [9].

This complexity creates difficulties in establishing a standardised definition of relevant terms and often only enables definitions at a general level. However, accurate and precise terminology is essential to ensure sufficient education, patient safety and high quality of care. Additionally, it enables standardisation of treatment and appropriate comparisons in research [10, 11, 12, 13, 14, 15, 16]. For instance, it concerns questions such as how CC services should be organised and staffed, which patient cohorts are defined as having a critical illness (CI), and when do they require CC.

Although terms such as ‘intensive care’ or ‘high dependency’ were previously used, the term ‘critical care’ has been introduced by the Department of Health of the United Kingdom in 2000 [17]. In their recommendation, they make it clear that the term focusses on the level of care that the patient requires, thereby decoupling the situation from the location. Mukherjee et al. recently confirmed this distinction; they state that CC is a concept and not a location [18].

Nevertheless, CC and CI are still defined heterogeneously [19, 20, 21], as are intensivists [22] or the scope of responsibilities for CC nurses [23]. The international scientific community still lacks a uniform definition of these key terms.

### 1.2 Aims

The aim of this review was to identify and critically discuss current definitions of CC, CI and chronic CI (CCI), and to analyse existing similarities and differences as well as to identify further research needs.

## 2 Design and Methods

In this regard, we designed a narrative review, following four steps: (1) define topic and audience, (2) search and re‐search the literature, (3) be critical and (4) find a logical structure, as recommended by Gregory et al. [24]. This design was particularly suitable because it enabled a discursive compilation of current literature.

### 2.1 Setting, Sample and Data Collection

First, we decided on the topic of interest, and discussed its scope and importance. Second, we developed an appropriate methodological approach characterised by several literature searches. In detail, we conducted formative searches in the databases Medline (via PubMed), Cochrane Library, Cumulative Index to Nursing and Allied Health Sciences (CINAHL, via EBSCO host), Embase (via Ovid), LIVIVO, Google Scholar, OpenGrey, Epistemonikos and Science Open [25]. In addition, we carried out citation searching [26]. The initial searches were performed from July 2024 to January 2025; an update of all searches was conducted in September 2025. The search strings are presented in Table S1. Reports on definitions were included in this review if they were available in German or English and had a recognisable thematic reference to one of the three main concepts (CC, CI, CCI). They also had to be published within the last 10 years (2015–2025) to identify and reflect on the current understanding as well as ongoing discussions about the definitions. No restrictions were made regarding the age of the patients, the setting or the development of the definitions.

### 2.2 Data Analysis

Third, we carried out analyses of the identified literature and its incorporated definitions. In this regard, we extracted data (authors, year, study design, definition and DOI/URL) from the sources into a predefined Excel sheet. The identified definitions were analysed inductively by two reviewers independently of each other. The derived themes were then compared, summarised into main concepts, merged into thematic clusters and discussed with a third reviewer in cases of inconsistencies. Finally, all clusters detected in this inductive thematic analysis were presented to the entire research group and discussed together [27]. Next to identifying core concepts of the investigated terminology, we also searched for research gaps and missing perspectives in these definitions. Fourth, we derived conclusions and developed recommendations following our critical discussion of the results.

In view of the lack of standardised reporting guidelines for narrative reviews, we oriented the reporting of this article along the methodological recommendations [24, 28] and the ‘Scale for the Quality Assessment of Narrative Review Articles’ (SANRA) [29]. The reporting of our abstract follows the current ‘Preferred Reporting Items for Systematic Reviews and Meta‐Analyses’ (PRISMA) guideline [30].

## 3 Results

In our formative database searches and additional literature searches, we identified 13 definitions of CC [19, 31, 32, 33, 34, 35, 36, 37, 38, 39, 40, 41, 42], eight definitions of CI [32, 35, 43, 44, 45, 46, 47, 48] and 12 definitions of CCI [49, 50, 51, 52, 53, 54, 55, 56, 57, 58, 59, 60]. These result from two concept analyses, 16 literature reviews, eight studies, one guideline and four websites. Following this, the included definitions vary in their evidence base. In addition, the breadth and scope of the underlying definitions differ significantly. The detailed study characteristics and the extracted definitions for the three key terms are presented in Table S2.

### 3.1 Definitions of ‘Critical Care’

Overall, we identified 13 definitions of CC resulting from two concept analyses [32, 35], five literature reviews [19, 33, 36, 40, 42], two primary studies [31, 41], one guideline [34] and three websites [37, 38, 39]. In the analysis, differences in their scope and descriptive breadth as well as several overlaps could be identified.

**FIGURE 1:**
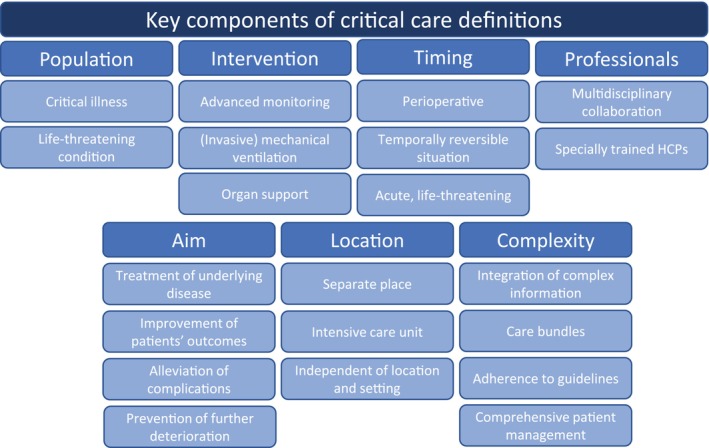
Key components of critical care definitions. The clusters identified during the analysis of identified definitions. HCP, healthcare professional.

A total of seven thematic clusters were formed in the analysis of the definitions included (Figure 1). Of the 13 definitions identified, 11 describe a specific ‘population’ targeted by CC. This usually involves people with CI [31, 34, 35, 37, 38, 41, 42] or a life‐threatening condition [33, 36, 39, 40]. A further eight definitions emphasise specific ‘interventions’ used in CC, for example, advanced monitoring [33, 37, 39, 40], invasive mechanical ventilation [31] and other organ support [33, 34, 35, 36, 40].

Eight definitions address ‘professionals’ in CC. Particular emphasis is placed on multidisciplinary collaboration [19, 33, 36, 42] between specially trained HCPs [32, 34, 39, 40]. CC also pursues various ‘aims’, which are specified in eight definitions. These include the treatment of underlying diseases [32, 34, 35, 36], the improvement of patients' outcomes [33], the alleviation of complications [32] and the prevention of further deterioration [36]. Seven definitions address the ‘location’ of CC. Six state that it is a separate place [33, 34, 37, 39, 40, 41], for example, the intensive care unit (ICU) [33, 34, 39, 40]. One emphasises that CC is independent of a location [42].

In addition, four definitions describe the ‘complexity’ of CC. They highlight the integration of complex information [33], care bundles [33], adherence to best (inter)national guidelines [34], comprehensive patient management [36] and overall specialised treatment [39]. Finally, five definitions mention the importance of ‘timing’. CC becomes relevant during the perioperative, temporally reversible situation [34] and is acute and life‐threatening in most cases [33, 36, 39, 40].

The analysis of the clusters reveals that different definitions emphasise various aspects. Although some definitions can only be found in two clusters [19, 31], other definitions have six [36] or seven [33, 34, 39] core aspects mentioned. Although the empirical and literature basis of the definitions differ along with the design of the studies, this has little significance in our analysis. Kayambankadzanja et al. [35] have considered a broad evidence base in their concept analysis, but only address the population, intervention and goal of CC in their brief definition. The literature reviews by Marshall et al. [36] and Jackson & Cairns [33] go far beyond this and address considerably more aspects in their longer definitions.

The definitions are primarily concerned with the underlying physiological impairments of patients and focus on interventions that are applied by specially trained HCPs for a specific purpose in a specific location. What is hardly considered is the experience of the patients and other people involved. There is only one definition highlighting that CC aims ‘to alleviate inherent physiopsychosocial complications’ [32].

### 3.2 Definitions of ‘Critical Illness’

**FIGURE 2:**
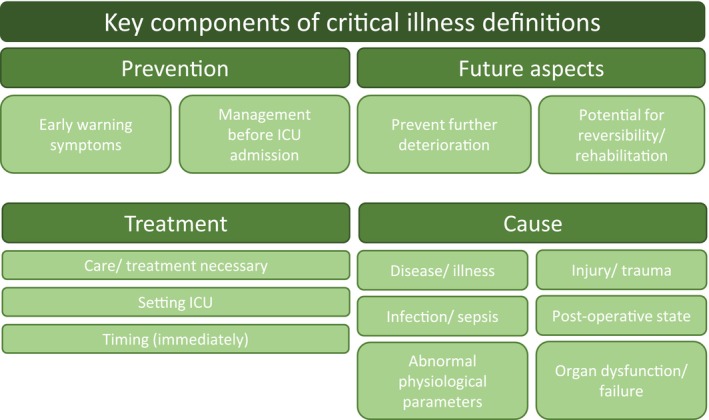
Key components of critical illness definitions. The clusters identified during the analysis of identified definitions. ICU, intensive care unit.

In total, we identified eight definitions of CI derived from two concept analyses [32, 35], four literature reviews [43, 45, 47, 48], one primary study [44] and one website [46] (Swiss Society for Intensive Care Medicine). When analysing the available definitions of CI, we identified a dynamic process, from which we were able to form four thematic clusters (Figure 2).

The first cluster deals with the aspect of ‘prevention’ of CI in two definitions by recognising early warning symptoms and (possibly) avoiding the necessity of an ICU admission.

In the second cluster, the 'causes' of CI from the definitions are summarised. Some of the causes mentioned in the studies are described basically as ‘disease/illness’ [43, 44, 46, 47], whereas more specific reasons such as ‘injury/trauma’ [43, 46, 47], 'infection/sepsis' [46, 47], ‘post‐operative state’ [43, 46, 47], ‘abnormal physiological parameters’ [43] or ‘organ dysfunction/failure’ [43, 46] are also presented.

Furthermore, the existing definitions deal with the ‘treatment’ of a CI, which is composed in the third cluster. In six definitions, the general ‘need for care/treatment’ is considered [32, 35, 43, 44, 46, 47]. Most of them describe the treatment as ‘immediately necessary’ [32, 43, 46] and primarily in the ‘ICU setting’ [44, 45, 46, 47, 48].

The fourth cluster summarises future aspects contained in the definitions. Two definitions describe the ‘prevention of further deterioration’ [43, 48], and another three characterise the 'potential for reversibility and rehabilitation' [35, 45, 48].

The identified definitions of CI show a temporal range from prevention to rehabilitation and indicate that CI starts even before intensive care becomes necessary and does not end with the ICU discharge. Ostermann and Vincent [45] described the CI as a continuous and dynamic sequence of interlinked events from the early moments of illness, through the stay in hospital/ICU and into recovery and rehabilitation. However, the temporal aspects before and after ICU admission are only considered in four studies [35, 43, 45, 48], whereas all definitions focus in some way on the cause and treatment of the CI. As with CC, the definitions of CI target the underlying physiological impairment of patients and the interventions performed. The experience of the CI or the experiences of patients and their relatives are not considered in any of the definitions.

### 3.3 Definitions of ‘Chronic Critical Illness’

**FIGURE 3:**
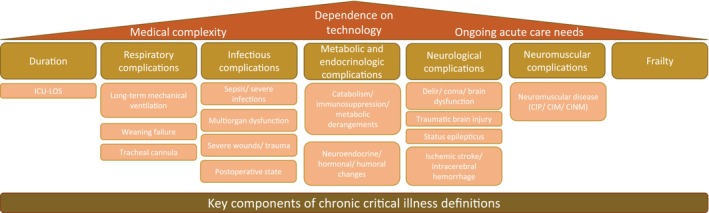
Key components of chronic critical illness definitions. The clusters identified during the analysis of identified definitions. CIM, critical illness myopathy; CINM, critical illness neuromyopathy; CIP, critical illness polyneuropathy; ICU, intensive care unit; LOS, length of stay.

We found 12 definitions of CCI; these result from seven reviews [49, 51, 53, 55, 56, 57, 60], four retrospective studies [50, 52, 58, 59] and one prospective cohort study [54]. Medical complexity, dependence on technology and ongoing care needs are overarching themes. Under this construction, we were able to abstract seven thematic clusters (Figure 3).

The first cluster deals with ICU length of stay (LOS). A similarity in all publications is a certain duration in ICU (beyond normal) [49, 50, 51, 52, 53, 54, 55, 56, 57, 58, 59, 60]; however, the minimum LOS in the ICU differs significantly, with durations varying from 72 h up to 21 days.

Nine out of 12 articles mention complications that are divided into five subsequent clusters. Prolonged mechanical ventilation, weaning failure or the presence of a tracheostomy are classified as ‘respiratory complications’ [49, 51, 53, 55, 56, 57, 60]. ‘Infectious complications’ encompass sepsis/serious infections, multiorgan dysfunction, severe wounds/trauma and post‐operative states [49, 51, 52, 55, 56, 59, 60].

‘Metabolic and endocrinologic complications’ include catabolism, immunosuppression, metabolic disturbances, as well as neuroendocrinological, humoral and hormonal changes [49, 51, 53, 56, 60]. Delirium, coma, brain dysfunction, traumatic brain injury, status epilepticus, ischaemic stroke and intracerebral haemorrhage are identified as ‘neurological complications’ [49, 51, 53, 55, 56, 60]. ‘Neuromuscular complications’ comprise CI polyneuropathy (CIP), CI myopathy (CIM) and CI neuromyopathy (CINM) [49, 51, 53, 56, 60].

The last cluster ‘frailty’ can be found in four definitions [49, 53, 56, 60]. It is characterised by a decline in the function of multiple physiological systems and an increased vulnerability to stressors. Frailty has been used to describe patients who have survived their initial CI and consequently a prolonged stay.

Looking at these clusters, CCI is defined by a certain length of time in the ICU combined with complications. These interfere with each other and create a complex medical situation that requires ongoing acute care, leaving the patient dependent on specialised technological support.

Iwashyna et al. [51] therefore postulate that patients with CCI are ICU‐dependent due to a cascade of CIs rather than because of their original ICU admitting diagnosis. CCI begins according to Shaw et al. [58] at the ‘point during an ICU stay when […] patients' acute diagnoses and physiologic disturbance are no longer more accurate at discriminating who survives than are baseline demographics and comorbidity’. As with CC and CI, the definitions of CCI focus on the (patho)physiological condition of the patient. The experience of the CCI, the relatives and the HCPs are not addressed by any of the definitions.

## 4 Discussion

The aim of this narrative review was to identify and discuss current definitions of CC, CI and CCI. In this regard, we conducted formative literature searches and identified 13 definitions of CC, eight definitions concerning CI and 12 definitions regarding CCI. Following an inductive analysis, we derived several clusters regarding the key components of these definitions.

The compilation of the clusters reveals both common themes and differences in the underlying definitions. One key aspect is ‘time’. Among all three subjects, a cluster was identified that emphasises temporality as an essential dimension. This is apparent in an urgent need for CC and evident in the continuum from CI to CCI. The scoping review by Ohbe et al. [61] also highlights the temporal dimension of CCI. Their 64 included definitions mainly referred to the LOS in ICU (≥ 8–21 days) or the duration of mechanical ventilation (≥ 7–14 days), pointing to the timing of CCI as a key factor, which was also found in this narrative review.

As with CC, both CI and CCI focus primarily on the cause and treatment of underlying problems. The definitions therefore address humans' physiology and make it clear that life is not possible without treatment; the ‘human system’ must be stabilised. In addition to the CI of physiology, the question arises as to whether a person can also be mentally critically ill. No information on this can be found in existing definitions. It remains unclear whether, for example, organ failure is always the result of a previous critical mental illness or whether it manifests itself differently.

Furthermore, the validity and transferability of these definitions are of great importance. The breadth and depth of CC and CI are always dependent on societal expectations and moral discussions. Analyses such as the prevalence and mortality in different countries [62, 63] or the 10/90 gap [64, 65] draw attention to an important aspect: CC and CI are defined in a theoretically similar way in different countries. The current African Critical Illness Outcomes Study (ACIOS) [66] examines CI in 22 African nations based on the definition of an international research group also identified in this review [35]. However, the results show that the resources for providing CC for CI differ significantly. Questions remain about health economy and health equity [67], which must also be considered in these definitions.

The focus in the definitions of CC, CI and CCI is on the inpatient setting, particularly on the ICU or the emergency department, as this is where time‐critical and immediate treatment is possible. Outpatient facilities were not excluded from this review, but the setting was not included in any of the identified definitions. This raises the question of further research considering whether CC, CI and CCI are tied to the inpatient setting or whether an outpatient‐clinical approach is also possible. A step in this direction can be observed in the example of long‐term acute care hospitals in the United States and must be considered in further discussions [68, 69, 70].

The transition from CI to CCI appears to be a theoretical construct. By the end of the CI, the patient has either recovered, died or remains in his/her situation (dependence on machines, instability, etc.). This last outcome, remaining in the situation, raises various questions: Is the CCI iatrogenic? Is mortality an outcome that can and should always be prevented? And what are the (long‐term) outcomes of CCI? Here, the discussion about overtreatment arises. Critical care is associated with ethical dilemmas and contrasts the questions of what can be done and what should be done [71, 72].

It becomes clear that the definitions of CC, CI and CCI are closely linked to the experiences of those affected. However, these links are still missing in the identified definitions. Although discussions and research have so far focussed on the trade‐off between life and death, survival could also be compared with patients' health‐related quality of life.

### 4.1 Limitations

This review has limitations. First, only definitions in German and English were included. Definitions in other languages were not considered in this review. Second, we only included definitions from the last 10 years to provide an up‐to‐date overview. The development of CC and older understandings are not sufficiently represented in this review. Third, we cannot ensure a comprehensive representation of all relevant definitions, as we have only carried out formative and not systematic searches. Fourth, no quality appraisal of included studies and definitions was conducted.

### 4.2 Implications for Clinical Practice

The differentiated analysis of the definitions is of great importance for HCPs, as they use them continuously in their everyday work. The clusters presented here can help to better reflect on and justify the differences. These clusters also help educators to convey the key characteristics of the setting and patients when training nursing staff. In addition, special attention should be paid to the experience of various groups, which offers the prospect of a contemporary and up‐to‐date definition.

### 4.3 Implications for Research

Further research should be based on international consensus, including countries, societies and health systems that have not yet been considered. Heterogeneous definitions emphasise the need for an internationally agreed understanding of the terms that adequately cover all relevant groups. It is therefore essential that definitions encompass the experiences of patients, relatives and HCPs, and that these are thoroughly reflected upon.

## 5 Conclusion

In summary, the definitions of CC, CI and CCI reveal a pathophysiological, medical focus (cause, diagnosis and treatment). Consequently, the experiences of those affected and human factors have hardly been incorporated into these definitions to date, although numerous studies are already available that depict and highlight the experiences of the various groups involved. This experience is heterogeneous; it differs from patient to patient and from situation to situation. After all, the objectives and feasibility of CC are fundamentally dependent on what the people involved experience and can contribute to the process.

## Author Contributions

All authors had a substantial contribution to the manuscript and approved it for submission.

## Funding

The authors have nothing to report.

## Ethics Statement

The authors have nothing to report.

## Consent

The authors have nothing to report. Permission to reproduce material from other sources and Clinical Trial Registration: Not applicable.

## Conflicts of Interest

The authors declare no conflicts of interest.

## Supporting information


**Table S1:** Search strategies.

**Table S2:** Characteristics and definitions of included sources.

## Data Availability

The data that support the findings of this study are available in the Supporting Information of this article.
