# Supplementary material for: A Reflection on Current Definitions of Critical Care and Critical Illness—A Narrative Review of the Literature
Source: Nurs Crit Care. 2026 Jan 8;31(1):e70311. doi: 10.1111/nicc.70311 (PMC12781964; doi:10.1111/nicc.70311)
Supplement: Supplementary file 1 — Table S1: Search strategies. [file NICC-31-0-s001.docx]

**Table S1 – Search strategies**

**Overview**

1. Search strategy on ‚Critical Care’ – p. 1-4
2. Search strategy on ‚Critical Illness’ – p. 5-6
3. Search strategy on ‚Chronic Critical Illness’ – p. 7-11

**Topic 1 – Definition ‘Critical Care’**

**Search strategies in systematic databases**

| **Initial search - Medline (via PubMed)** | | | | |
| --- | --- | --- | --- | --- |
| **Nr.** | **Search string** | **Filter** | **Results** | **Date of search** |
| 01 | ((((((((((defin*[Title/Abstract]) OR (terminolog*[Title/Abstract])) OR (characteri*[Title/Abstract])) OR (concept*[Title/Abstract])) OR (explain*[Title/Abstract])) OR (explan*[Title/Abstract])) OR (understand*[Title/Abstract])) OR (associat*[Title/Abstract])) OR (mean*[Title/Abstract])) OR (descri*[Title/Abstract])) AND (((((critical care[MeSH Terms]) OR (critical care[Title/Abstract])) OR (acute care[Title/Abstract])) OR (urgent care[Title/Abstract])) OR (intensive care[Title/Abstract])) | Last 10 years | 106,326 | 07/29/2024 |
| 02 | ((((((((((defin*[Title/Abstract]) OR (terminolog*[Title/Abstract])) OR (characteri*[Title/Abstract])) OR (concept*[Title/Abstract])) OR (explain*[Title/Abstract])) OR (explan*[Title/Abstract])) OR (understand*[Title/Abstract])) OR (associat*[Title/Abstract])) OR (mean*[Title/Abstract])) OR (descri*[Title/Abstract])) AND ((critical care[MeSH Terms]) OR (critical care[Title/Abstract])) | Last 10 years | 26,905 | 07/29/2024 |
| 03 | (((defin*[Title/Abstract]) OR (terminolog*[Title/Abstract])) OR (concept*[Title/Abstract])) AND ((critical care[MeSH Terms]) OR (critical care[Title/Abstract])) | Last 10 years | 5,762 | 07/29/2024 |
| 04 | definition critical care | Last 10 years | 7,888 | 08/26/2024 |
| 05 | terminology critical care | Last 10 years | 916 | 09/26/2024 |
| **Updated search - Medline (via PubMed)** | | | | |
| **Nr.** | **Search string** | **Filter** | **Results** | **Date of search** |
| 01 | ((((((((((defin*[Title/Abstract]) OR (terminolog*[Title/Abstract])) OR (characteri*[Title/Abstract])) OR (concept*[Title/Abstract])) OR (explain*[Title/Abstract])) OR (explan*[Title/Abstract])) OR (understand*[Title/Abstract])) OR (associat*[Title/Abstract])) OR (mean*[Title/Abstract])) OR (descri*[Title/Abstract])) AND (((((critical care[MeSH Terms]) OR (critical care[Title/Abstract])) OR (acute care[Title/Abstract])) OR (urgent care[Title/Abstract])) OR (intensive care[Title/Abstract])) | July 2024 – August 2025 | 19,446 | 09/02/2025 |
| 02 | ((((((((((defin*[Title/Abstract]) OR (terminolog*[Title/Abstract])) OR (characteri*[Title/Abstract])) OR (concept*[Title/Abstract])) OR (explain*[Title/Abstract])) OR (explan*[Title/Abstract])) OR (understand*[Title/Abstract])) OR (associat*[Title/Abstract])) OR (mean*[Title/Abstract])) OR (descri*[Title/Abstract])) AND ((critical care[MeSH Terms]) OR (critical care[Title/Abstract])) | July 2024 – August 2025 | 4,503 | 09/02/2025 |
| 03 | (((defin*[Title/Abstract]) OR (terminolog*[Title/Abstract])) OR (concept*[Title/Abstract])) AND ((critical care[MeSH Terms]) OR (critical care[Title/Abstract])) | July 2024 – August 2025 | 923 | 09/02/2025 |
| 04 | definition critical care | July 2024 – August 2025 | 1,642 | 09/02/2025 |
| 05 | terminology critical care | July 2024 – August 2025 | 234 | 09/02/2025 |

| **Initial search - Cochrane Library** | | | | |
| --- | --- | --- | --- | --- |
| **Nr.** | **Search string** | **Filter** | **Results** | **Date of search** |
| 01 | Defin* (TI,AB,KW) | None | 159,728 | 08/26/2024 |
| 02 | Critical care (TI,AB,KW) | None | 15,284 | 08/26/2024 |
| 03 | Defin* AND Critical care (TI,AB,KW) | None | 2,045 | 08/26/2024 |
| 04 | Defin* AND Critical care (TI,AB,KW) | Last 10 years | 1,740 | 08/26/2024 |
| 05 | Definition critical care | None | 1,965 | 08/26/2024 |
| 06 | Definition critical care | Last 10 years | 1,541 | 08/26/2024 |
| 07 | terminology critical care | None | 361 | 09/26/2024 |
| 08 | Terminology* AND critical care | None | 372 | 09/26/2024 |

| **Updated search - Cochrane Library** | | | | |
| --- | --- | --- | --- | --- |
| **Nr.** | **Search string** | **Filter** | **Results** | **Date of search** |
| 01 | Defin* (TI,AB,KW) | July 2024 – August 2025 | 115 | 09/02/2025 |
| 02 | Critical care (TI,AB,KW) | July 2024 – August 2025 | 51 | 09/02/2025 |
| 03 | Defin* AND Critical care (TI,AB,KW) | July 2024 – August 2025 | 51 | 09/02/2025 |
| 04 | Definition critical care | July 2024 – August 2025 | 232 | 09/02/2025 |
| 05 | terminology critical care | July 2024 – August 2025 | 42 | 09/02/2025 |
| 06 | Terminology* AND critical care | July 2024 – August 2025 | 43 | 09/02/2025 |

| **Initial search - CINAHL (via EBSCO host)** | | | | |
| --- | --- | --- | --- | --- |
| **Nr.** | **Search string** | **Filter** | **Results** | **Date of search** |
| 01 | (definition or define or meaning or description) AND (critical care) | Last 10 years | 1,593 | 07/29/2024 |
| 02 | definition critical care | None | 51 | 08/26/2024 |
| 03 | Terminology critical care | Last 10 years | 15 | 09/26/2024 |
| 04 | Terminolog* AND critical care | Last 10 years | 112 | 09/26/2024 |

| **Updated search - CINAHL (via EBSCO host)** | | | | |
| --- | --- | --- | --- | --- |
| **Nr.** | **Search string** | **Filter** | **Results** | **Date of search** |
| 01 | (definition or define or meaning or description) AND (critical care) | July 2024 – August 2025 | 176 | 09/02/2025 |
| 02 | definition critical care | July 2024 – August 2025 | 1 | 09/02/2025 |
| 03 | Terminology critical care | July 2024 – August 2025 | 0 | 09/02/2025 |
| 04 | Terminolog* AND critical care | July 2024 – August 2025 | 9 | 09/02/2025 |

| **Initial search - Embase (via Ovid)** | | | | |
| --- | --- | --- | --- | --- |
| **Nr.** | **Search string** | **Filter** | **Results** | **Date of search** |
| 01 | (definition or define or meaning or description or terminology*) | Last 10 years | 1,573,334 | 09/23/2024 |
| 02 | Critical care | Last 10 years | 178,523 | 09/23/2024 |
| 03 | (definition or define or meaning or description or terminology*) AND (critical care) | Last 10 years | 40,537 | 09/23/2024 |
| 04 | Terminology critical care | None | 14 | 09/23/2024 |
| 05 | Definition critical care | None | 79 | 09/23/2024 |

| **Updated search - Embase (via Ovid)** | | | | |
| --- | --- | --- | --- | --- |
| **Nr.** | **Search string** | **Filter** | **Results** | **Date of search** |
| 01 | (definition or define or meaning or description or terminology*) | July 2024 – August 2025 | 78,055 | 09/02/2025 |
| 02 | Critical care | July 2024 – August 2025 | 2,378 | 09/02/2025 |
| 03 | (definition or define or meaning or description or terminology*) AND (critical care) | July 2024 – August 2025 | 871 | 09/02/2025 |
| 04 | Terminology critical care | July 2024 – August 2025 | 1 | 09/02/2025 |
| 05 | Definition critical care | July 2024 – August 2025 | 4 | 09/02/2025 |

**Additional searches**

| **Source** | **Search string** | **Filter** | **Results** | **Date of search** |
| --- | --- | --- | --- | --- |
| Google Scholar | Definition critical care | Last 10 years | 17,800 | 07/29/2024 |
|  | Terminology critical care | Last 10 years | 193,000 | 09/23/2024 |
| LIVIVO | Definition critical care | Last 10 years | 10,186 | 07/29/2024 |
|  | Terminology critical care | Last 10 years | 697 | 09/23/2024 |
| OpenGrey | Definition critical care | None | 0 | 07/29/2024 |
|  | Terminology critical care | None | 0 | 09/23/2024 |
| Epistemonikos | Definition critical care | None | 13 | 09/23/2024 |
|  | Terminology critical care | None | 58 | 09/23/2024 |
| Science Open | Definition critical care | None | 0 | 09/23/2024 |
|  | Terminology critical care | None | 0 | 09/23/2024 |

**Backwards citation searching**

**Seed references:**

- In: Benneyworth et al. (2015)
- In: Christensen & Liang (2023)
- In: Crawford et al. (2023)
- In: Kayambankadzanja et al. (2022)
- In: Marshall et al. (2017)
- In: Tanner & Cornish (2020)
- In: Vasco 2019

**Forward citation searching**

**Seed references:**

- With: Christensen & Liang (2023)
- With: Kayambankadzanja et al. (2022)

**Topic 2 – Definition ‘Critical Illness’**

**Search strategies in systematic databases**

| **Initial search - Medline (via PubMed)** | | | | |
| --- | --- | --- | --- | --- |
| **Nr.** | **Search string** | **Filter** | **Results** | **Date of search** |
| 01 | ((definit*[Title]) OR (terminolog*[Title])) AND (critical illness[Title/Abstract]) | Last 10 years | 22 | 07/24/2024 |
| 02 | "critical illness"[Title/Abstract] AND "definition"[Title/Abstract] | Last 10 years | 165 | 07/25/2024 |

| **Updated search - Medline (via PubMed)** | | | | |
| --- | --- | --- | --- | --- |
| **Nr.** | **Search string** | **Filter** | **Results** | **Date of search** |
| 01 | ((definit*[Title]) OR (terminolog*[Title])) AND (critical illness[Title/Abstract]) | July 2024 – August 2025 | 7 | 09/03/2025 |
| 02 | "critical illness"[Title/Abstract] AND "definition"[Title/Abstract] | July 2024 – August 2025 | 20 | 09/03/2025 |

| **Initial search - Cochrane Library** | | | | |
| --- | --- | --- | --- | --- |
| **Nr.** | **Search string** | **Filter** | **Results** | **Date of search** |
| 01 | TI, AB, KW Definition critical illness | Last 10 years | 20 | 08/29/2024 |
| 02 | MeSH Critical Illness AND Ti, AB, KW Definition | Last 10 years | 3 | 08/29/2024 |

| **Updated search - Cochrane Library** | | | | |
| --- | --- | --- | --- | --- |
| **Nr.** | **Search string** | **Filter** | **Results** | **Date of search** |
| 01 | TI, AB, KW Definition critical illness | July 2024 – August 2025 | 21 | 09/03/2025 |
| 02 | MeSH Critical Illness AND Ti, AB, KW Definition | July 2024 – August 2025 | 14 | 09/03/2025 |

| **Initial search - CINAHL (via EBSCO host)** | | | | |
| --- | --- | --- | --- | --- |
| **Nr.** | **Search string** | **Filter** | **Results** | **Date of search** |
| 01 | TI critical illness OR AB critical illness AND TI definition OR AB definition | Last 10 years | 97 | 08/29/2024 |
| 02 | Definition critical illness | None | 4 | 09/24/2024 |

| **Updated search - CINAHL (via EBSCO host)** | | | | |
| --- | --- | --- | --- | --- |
| **Nr.** | **Search string** | **Filter** | **Results** | **Date of search** |
| 01 | TI critical illness OR AB critical illness AND TI definition OR AB definition | July 2024 – August 2025 | 7 | 09/03/2025 |
| 02 | Definition critical illness | July 2024 – August 2025 | 1 | 09/03/2025 |

**Additional searches**

| **Source** | **Search string** | **Filter** | **Results** | **Date of search** |
| --- | --- | --- | --- | --- |
| Google Scholar | Critical illness definition | Last 10 years | 17.900 | 07/25/2024 |
|  | Terminology critical illness | Last 10 years | 30.300 | 09/24/2024 |
| Livivo | Definition critical illness | Last 10 years | 1.495 | 09/24/2024 |
|  | Terminology critical illness | Last 10 years | 111 | 09/24/2024 |
| Open Grey | Definition critical illness | None | 0 | 09/24/2024 |
|  | Terminology critical illness | None | 0 | 09/24/2024 |

**Backwards citation searching**

**Seed references:**

- In: Kayambankazania et al. (2022)
- In: Cristensen & Liang (2023)
- In: Arias et al. (2024)
- In: Maslove et al. (2022)
- In: Sharma et al. (2019)
- In: Ostermann & Vinvent (2023)
- In: Vincent (2019)

**Forward citation searching**

**Seed references:**

- With: Kayambankazania et al. (2022)
- With: Cristensen & Liang (2023)
- With: Arias et al. (2024)
- With Maslove et al. (2022)
- With: Sharma et al. (2019)
- With: Ostermann & Vinvent (2023)
- With: Vincent (2019)

**Topic 3 – Definition ‘Chronic Critical Illness’**

**Search strategies in systematic databases**

| **Initial search - Medline (via PubMed)** | | | | |
| --- | --- | --- | --- | --- |
| **Nr.** | **Search string** | **Filter** | **Results** | **Date of search** |
| 01 | Chronic* critic* ill*[Title/Abstract] | None | 514 | 07/28/2024 |
| 02 | Prolong* critic* ill*[Title/Abstract] | None | 182 | 07/28/2024 |
| 03 | Persistent critic* ill*[Title/Abstract] | None | 49 | 07/28/2024 |
| 04 | “persistent inflammation immunosuppression and catabolism syndrome” Title/Abstract] | None | 73 | 07/28/2024 |
| 05 | “long stay intensive care unit patient” [Title/Abstract] – Schema: all | None | 0 | 07/28/2024 |
| 06 | “long stay intensive care unit patient” [Title/Abstract] | None | 0 | 07/28/2024 |
| 07 | “long stay intensive care unit patients” [Title/Abstract] | None | 5 | 07/28/2024 |
| 08 | Ongoing critic* ill*[Title/Abstract] | None | 1,408 | 07/28/2024 |
| 09 | “Ongoing critic* ill*”[Title/Abstract] – Schema: all | None | 0 | 07/28/2024 |
| 10 | “ongoing critic* ill*”[Title/Abstract] | None | 0 | 07/28/2024 |
| 11 | Chronic* persistent* ill*[Title/Abstract] | None | 36 | 07/28/2024 |
| 12 | Prolonged persistent* ill*[Title/Abstract] | None | 6 | 07/28/2024 |
| 13 | Chronic* persistent* major ill*[Title/Abstract] | None | 5 | 07/28/2024 |
| 14 | Prolonged persistent major ill*[Title/Abstract] | None | 1 | 07/28/2024 |
| 15 | Chronic* persistent organ fail*[Title/Abstract] | None | 31 | 07/28/2024 |
| 16 | Chronic persistent severe diseas*[Title/Abstract] | None | 5 | 07/28/2024 |
| 17 | Prolonged persistent severe disease*[Title/Abstract] – Schema: all | None | 0 | 07/28/2024 |
| 18 | Prolonged persistent severe diseas*[Title/Abstract] | None | 0 | 07/28/2024 |
| 19 | Chronic persistent critical* disorder*[Title/Abstract] | None | 2 | 07/28/2024 |
| 20 | Prolonged persistent critical* disorder[Title/Abstract] – Schema: all | None | 0 | 07/28/2024 |
| 21 | Prolonged persistent critical* disorder[Title/Abstract] | None | 0 | 07/28/2024 |
| 22 | Chronic persistent severe disorder*[Title/Abstract] | None | 12 | 07/28/2024 |
| 23 | Prolonged persistent severe disorder*[Title/Abstract] | None | 5 | 07/28/2024 |
| 24 | Chronic persistent disorder*[Title/Abstract] | None | 23 | 07/28/2024 |
| 25 | Prolonged persistent disorder*[Title/Abstract] | None | 3 | 07/28/2024 |
| 26 | Chronic persistent major disorder*[Title/Abstract] | None | 2 | 07/28/2024 |
| 27 | Prolonged persistent major disorder*[Title/Abstract] – Schema: all | None | 0 | 07/28/2024 |
| 28 | prolonged persistent major disorder*[Title/Abstract] |  | 0 | 07/28/2024 |

| **Updated search - Medline (via PubMed)** | | | | |
| --- | --- | --- | --- | --- |
| **Nr.** | **Search string** | **Filter** | **Results** | **Date of search** |
| 01 | Chronic* critic* ill*[Title/Abstract] | July 2024 – August 2025 | 68 | 09/03/2025 |
| 02 | Prolong* critic* ill*[Title/Abstract] | July 2024 – August 2025 | 7 | 09/03/2025 |
| 03 | Persistent critic* ill*[Title/Abstract] | July 2024 – August 2025 | 20 | 09/03/2025 |
| 04 | “persistent inflammation immunosuppression and catabolism syndrome” Title/Abstract] | July 2024 – August 2025 | 0 | 09/03/2025 |
| 05 | “long stay intensive care unit patient” [Title/Abstract] – Schema: all | July 2024 – August 2025 | 0 | 09/03/2025 |
| 06 | “long stay intensive care unit patient” [Title/Abstract] | July 2024 – August 2025 | 0 | 09/03/2025 |
| 07 | “long stay intensive care unit patients” [Title/Abstract] | July 2024 – August 2025 | 0 | 09/03/2025 |
| 08 | Ongoing critic* ill*[Title/Abstract] | July 2024 – August 2025 | 172 | 09/03/2025 |
| 09 | “Ongoing critic* ill*”[Title/Abstract] – Schema: all | July 2024 – August 2025 | 0 | 09/03/2025 |
| 10 | “ongoing critic* ill*”[Title/Abstract] | July 2024 – August 2025 | 0 | 09/03/2025 |
| 11 | Chronic* persistent* ill*[Title/Abstract] | July 2024 – August 2025 | 4 | 09/03/2025 |
| 12 | Prolonged persistent* ill*[Title/Abstract] | July 2024 – August 2025 | 0 | 09/03/2025 |
| 13 | Chronic* persistent* major ill*[Title/Abstract] | July 2024 – August 2025 | 0 | 09/03/2025 |
| 14 | Prolonged persistent major ill*[Title/Abstract] | July 2024 – August 2025 | 0 | 09/03/2025 |
| 15 | Chronic* persistent organ fail*[Title/Abstract] | July 2024 – August 2025 | 3 | 09/03/2025 |
| 16 | Chronic persistent severe diseas*[Title/Abstract] | July 2024 – August 2025 | 0 | 09/03/2025 |
| 17 | Prolonged persistent severe disease*[Title/Abstract] – Schema: all | July 2024 – August 2025 | 0 | 09/03/2025 |
| 18 | Prolonged persistent severe diseas*[Title/Abstract] | July 2024 – August 2025 | 0 | 09/03/2025 |
| 19 | Chronic persistent critical* disorder*[Title/Abstract] | July 2024 – August 2025 | 0 | 09/03/2025 |
| 20 | Prolonged persistent critical* disorder[Title/Abstract] – Schema: all | July 2024 – August 2025 | 0 | 09/03/2025 |
| 21 | Prolonged persistent critical* disorder[Title/Abstract] | July 2024 – August 2025 | 0 | 09/03/2025 |
| 22 | Chronic persistent severe disorder*[Title/Abstract] | July 2024 – August 2025 | 0 | 09/03/2025 |
| 23 | Prolonged persistent severe disorder*[Title/Abstract] | July 2024 – August 2025 | 0 | 09/03/2025 |
| 24 | Chronic persistent disorder*[Title/Abstract] | July 2024 – August 2025 | 1 | 09/03/2025 |
| 25 | Prolonged persistent disorder*[Title/Abstract] | July 2024 – August 2025 | 0 | 09/03/2025 |
| 26 | Chronic persistent major disorder*[Title/Abstract] | July 2024 – August 2025 | 0 | 09/03/2025 |
| 27 | Prolonged persistent major disorder*[Title/Abstract] – Schema: all | July 2024 – August 2025 | 0 | 09/03/2025 |
| 28 | prolonged persistent major disorder*[Title/Abstract] | July 2024 – August 2025 | 0 | 09/03/2025 |

| **Initial search - Cochrane Library** | | | | |
| --- | --- | --- | --- | --- |
| **Nr.** | **Search string** | **Filter** | **Results** | **Date of search** |
| 01 | ("Persistent inflammation, immunosuppression and catabolism syndrome"):ti,ab,kw | None | 5 | 06/10/2024 |
| 02 | ("persistent critical illness"):ti,ab,kw | None | 2 | 06/10/2024 |
| 03 | ("Chronic critical illness"):ti,ab,kw (Word variations have been searched) | None | 41 | 06/10/2024 |

| **Updated search - Cochrane Library** | | | | |
| --- | --- | --- | --- | --- |
| **Nr.** | **Search string** | **Filter** | **Results** | **Date of search** |
| 01 | ("Persistent inflammation, immunosuppression and catabolism syndrome"):ti,ab,kw | July 2024 – August 2025 | 0 | 09/03/2025 |
| 02 | ("persistent critical illness"):ti,ab,kw | July 2024 – August 2025 | 0 | 09/03/2025 |
| 03 | ("Chronic critical illness"):ti,ab,kw | July 2024 – August 2025 | 1 | 09/03/2025 |

| **Initial search - CINAHL (via EBSCO host)** | | | | |
| --- | --- | --- | --- | --- |
| **Nr.** | **Search string** | **Filter** | **Results** | **Date of search** |
| 01 | TI chronic* OR AB chronic* | None | 322,069 | 06/10/2024 |
| 02 | TI critic* OR AB critic* | None | 232,518 | 06/10/2024 |
| 03 | TI ill* OR AB ill* | None | 265,694 | 06/10/2024 |
| **04** | **01 AND 02 AND 03** | None | 3,765 | 06/10/2024 |
| 05 | TI chronic critical illness OR AB chronic critical illness | None | 273 | 06/10/2024 |
| 06 | TI (definition or define or meaning or description) OR AB (definition or define or meaning or description) | None | 194,877 | 06/10/2024 |
| **07** | **05 AND 06** | None | 26 | 06/10/2024 |
| **08** | **05 AND 06** | Last 10 years | 18 | 06/10/2024 |

| **Updated search - CINAHL (via EBSCO host)** | | | | |
| --- | --- | --- | --- | --- |
| **Nr.** | **Search string** | **Filter** | **Results** | **Date of search** |
| 01 | TI chronic* OR AB chronic* | July 2024 – August 2025 | 17,479 | 09/03/2025 |
| 02 | TI critic* OR AB critic* | July 2024 – August 2025 | 18,163 | 09/03/2025 |
| 03 | TI ill* OR AB ill* | July 2024 – August 2025 | 265,694 | 09/03/2025 |
| **04** | **01 AND 02 AND 03** | July 2024 – August 2025 | 198 | 09/03/2025 |
| 05 | TI chronic critical illness OR AB chronic critical illness | July 2024 – August 2025 | 15 | 09/03/2025 |
| 06 | TI (definition or define or meaning or description) OR AB (definition or define or meaning or description) | July 2024 – August 2025 | 10,403 | 09/03/2025 |
| **07** | **05 AND 06** | July 2024 – August 2025 | 2 | 09/03/2025 |

| **Initial search - Embase (via Ovid)** | | | | |
| --- | --- | --- | --- | --- |
| **Nr.** | **Search string** | **Filter** | **Results** | **Date of search** |
| 01 | Chronic* critic* ill*.mp.[mp=tx, bt, ti, ab, ct] | None | 1,317 | 16/10/2024 |
| 02 | “chronic* critic* ill*”.m_titl. | None | 183 | 16/10/2024 |
| 03 | (defin* or mean* or descri* or terminol*).mp. [mp=tx, bt, ti, ab, ct] | None | 7,692,934 | 16/10/2024 |
| 04 | 01 AND 03 | None | 1205 | 16/10/2024 |
| 05 | 02 AND 03 | None | 161 | 16/10/2024 |
| 06 | (defin* or mean* or descri* or terminol*).m_titl. | None | 123,724 | 16/10/2024 |
| 07 | 02 AND 06 | None | 6 | 16/10/2024 |
| 08 | 02 AND 03 | Last 10 years | 111 | 16/10/2024 |

| **Updated search - Embase (via Ovid)** | | | | |
| --- | --- | --- | --- | --- |
| **Nr.** | **Search string** | **Filter** | **Results** | **Date of search** |
| 01 | Chronic* critic* ill*.mp.[mp=tx, bt, ti, ab, ct] | July 2024 – August 2025 | 49 | 09/03/2025 |
| 02 | “chronic* critic* ill*”.m_titl. | July 2024 – August 2025 | 17 | 09/03/2025 |
| 03 | (defin* or mean* or descri* or terminol*).mp. [mp=tx, bt, ti, ab, ct] | July 2024 – August 2025 | 87,632 | 09/03/2025 |
| 04 | 01 AND 03 | July 2024 – August 2025 | 48 | 09/03/2025 |
| 05 | 02 AND 03 | July 2024 – August 2025 | 3 | 09/03/2025 |
| 06 | (defin* or mean* or descri* or terminol*).m_titl. | July 2024 – August 2025 | 4,003 | 09/03/2025 |
| 07 | 02 AND 06 | July 2024 – August 2025 | 0 | 09/03/2025 |
| 08 | 02 AND 03 | July 2024 – August 2025 | 8 | 09/03/2025 |

**Additional searches**

| **Source** | **Search string** | **Filter** | **Results** | **Date of**  **search** |
| --- | --- | --- | --- | --- |
| Google  Scholar | "chronic critical illness" + "definition" | Last 10 years | 1900 | 10/082024 |
|  | "chronically critically ill" + "definition" | Last 10 years | 906 |  |
|  | “CCI” + "definition" | Last 10 years | 27,800 |  |
| LIVIVO | chronic* critic ill* AND defin* | Last 10 years | 1147 | 09/30/2024 |
| OpenGrey | "chronic critical illness" + "definition" | None | 0 | 09/30/2024 |
|  | "chronically critically ill" + "definition" | None | 0 |  |
|  | "chronic critical illness" | None | 0 |  |
|  | "chronically critically ill" | None | 0 |  |
| Epistemonikos | title:(chronic* critic* ill*) OR abstract:(chronic* critic* ill*) | Last 10 years | 1789 | 09/30/2024 |
|  | title:(defin*) OR title:("terminol") | Last 10 years | 5408 |  |
|  | title:((chronic) OR (chronical) OR (chronically)) AND title:((critic) OR (critical) OR (critically)) AND title:((ill) OR (illness)) | Last 10 years | 66 |  |
|  | title:((chronic) OR (chronical) OR (chronically)) AND title:((critic) OR (critical) OR (critically)) AND title:((ill) OR (illness)) AND title:(defin*) OR title:("terminol") | Last 10 years | 1 |  |
| Science Open | "chronic*" "critic*" "ill*" | Last 10 years | 377 | 10/10/2024 |
|  |  | Title | 221 |  |
|  |  | Narrow by Key word “chronic critical illness” | 36 |  |
|  | definition AND "chronic critical illness" | Last 10 years | 33 |  |
