# Supplementary material for: A Reflection on Current Definitions of Critical Care and Critical Illness—A Narrative Review of the Literature
Source: Nurs Crit Care. 2026 Jan 8;31(1):e70311. doi: 10.1111/nicc.70311 (PMC12781964; doi:10.1111/nicc.70311)
Supplement: Supplementary file 2 — Table S2: Characteristics and definitions of included sources. [file NICC-31-0-s002.docx]

**Table S2: Characteristics and definitions of included sources**

**Overview:**

- Definitions of critical care – pages 1-2
- Definitions of critical illness – pages 3-4
- Definitions of chronic critical illness – pages 5-6

**Table 1: Definitions of critical care**

| **Nr.** | **Author** | **Year** | **Study design (if available)** | **Definition** | **DOI/ Link** |
| --- | --- | --- | --- | --- | --- |
| 01 | Benneyworth et al. | 2015 | Retrospective data analysis | CC services were defined by the presence of an ICD-9-CM code for cardiac or pulmonary  arrest (799.1), respiratory failure (518.8×), apnea (786.03), or delivery of invasive mechanical ventilation (96.7×). | https://doi.org/10.1186/s13104-015-1550-9 |
| 02 | Christensen & Liang | 2023 | Concept analysis | Critical care is the application of advanced medical technologies administered by specialist health care professionals to alleviate the inherent physiopsychosocial complications associated with critical illness while treating the underlying disease process.  From this definition, the characteristics of critical care encompass the three health professions based on their respective expertise in supporting critical illness. Medicine as supporting and treating the underlying pathophysiology - the person as a disease process. Nursing as the administrators of care cooperating and supporting respective treatment options as well as applying their own specialised nursing care - the patient as a person. Allied health as the ‘rebuilders’ - the patient as structurally dysfunctional. | https://doi.org/10.1016/j.ijnss.2023.06.020 |
| 03 | Crawford et al. | 2023 | Literature review | Critical care requires a balance between meaningful survival and utilization of scare resources. It is best provided by a well-trained multidisciplinary team. | https://doi.org/10.1186/s13054-022-04296-3 |
| 04 | The International Surgical Outcomes Study group | 2016 | Prospective cohort study | Critical care is a facility routinely capable of admitting patients who require invasive ventilation overnight. | https://doi.org/10.1093/bja/aew316 |
| 05 | Jackson & Cairns | 2020 | Literature review | Critical care is the process of looking after patients who either suffer from life-threatening conditions or are at risk of developing them. The intensive care unit (ICU) is a distinct geographical entity in which high staffing ratios, advanced monitoring and organ support can be offered to improve patient morbidity and mortality. However, effective intensive care demands an integrated approach that stretches beyond the boundaries of the ICU. It requires prevention, early warning and response systems, a multidisciplinary approach before and during an ICU stay, as well as comprehensive follow-up or good quality palliative care.  Comprehensive care for critically ill patients usually requires a systems-based approach and integration of complex information. To provide a consistently high standard of care, some interventions have been grouped into ‘care bundles’, which have been shown to improve outcome when implemented together. | 10.1016/j.mpsur.2020.11.002 |
| 06 | Joint Faculty of Intensive Care Medicine of Ireland | 2019 | National standard | Critical Care Units provide life sustaining treatment for critically ill patients with  acute organ dysfunction due to potentially reversible disease. It is the purpose of  the Unit to support the patient’s failing organs and diagnose and treat the  underlying cause. Patients at risk of organ dysfunction due to chronic disease  processes may also benefit from critical care in the peri-operative period or other  temporary reversible circumstance e.g. after trauma.  A Critical Care Service comprises appropriately trained and accredited medical,  nursing and allied health professionals based in a Critical Care Unit (see below),  working within a quality and governance structure consistent with delivery of the  best critical care while adhering to national and international best practice  guidelines. | https://jficmi.anaesthesia.ie/wp-content/uploads/2019/09/National-Standards-for-Adult-Critical-Services-2019.pdf |
| 07 | Kayambankadzanja et al. | 2022 | Concept analysis | Critical care is the identification, monitoring, and treatment of patients with critical illness through the initial and sustained support of vital organ functions. | https://doi.org/10.1136/bmjopen-2022-060972 |
| 08 | Marshall et al. | 2017 | Scoping review | Intensive care, also known as critical care, is a multidisciplinary and interprofessional specialty dedicated to the comprehensive management of patients having, or at risk of developing, acute, lifethreatening organ dysfunction. Intensive care uses an array of technologies that provide support of failing organ systems, particularly the lungs, cardiovascular system, and kidneys. Although the specialty has developed expertise in the comprehensive management of disorders such as sepsis and the acute respiratory distress syndrome, its common expertise is the pathophysiology and support of organ dysfunction more than the specific management of the diseases responsible for the acute illness; the primary goal of intensive care is to prevent further physiologic deterioration while the underlying disease is treated and resolves. | https://doi.org/10.1016/j.jcrc.2016.07.015 |
| 09 | Merriam-Webster | 2024 | Homepage | 1) the monitoring and treatment of critically ill or injured patients  2) a designated area of a hospital providing critical care | https://www.merriam-webster.com/dictionary/critical%20care |
| 10 | National Library of Medicine | 2024 | Homepage | Critical care is medical care for people who have life-threatening injuries and illnesses. It usually takes place in an intensive care unit (ICU). A team of specially-trained health care providers gives you 24-hour care. This includes using machines to constantly monitor your vital signs. It also usually involves giving you specialized treatments. | https://medlineplus.gov/criticalcare.html |
| 11 | National Library of Medicine | 2023 | Homepage | Health care provided to a critically ill patient during a medical emergency or crisis. | https://www.ncbi.nlm.nih.gov/mesh/68003422 |
| 12 | Tanner & Cornish | 2020 | Systematic review and meta-analysis | Critical care units provide care to the most unwell patients, who require specialist treatment because of life-threatening conditions. They are able to provide continuous monitoring and support for these patients because they have: higher staffing levels than general wards, staff with specific critical care expertise, advanced technology, and aggressive interventions, which are generally not available elsewhere. | https://doi.org/10.1111/nicc.12572 |
| 13 | Vasco | 2019 | Narrative review | ‘‘Maternal Critical Care” describes the interdisciplinary care of any pregnant patient, according to the severity of their medical condition, regardless of the location in a hospital. | https://doi.org/10.1016/j.ijoa.2018.09.010 |

**Table 2: Definitions of critical illness**

| **Nr.** | **Author** | **Year** | **Study design (if available)** | **Definition** | **DOI/ Link** |
| --- | --- | --- | --- | --- | --- |
| 01 | Arias et al. | 2024 | Scoping review followed by Delphi consensus | Definition for acute pediatric critical illness (DEFCRIT): “an infant, child or adolescent with an illness, injury, or post-operative state that increases the risk for or results in acute physiological instability (abnormal physiological parameters or vital organ dysfunction or failure) or a clinical support requirement (such as frequent or continuous monitoring or time-sensitive interventions) to prevent further deterioration or death” | 10.1016/S2214-109X(23)00537-5 |
| 02 | Christensen & Liang | 2023 | Concept analysis | Critical illness can be considered along a spectrum of adaption to complete organ failure with maladaptation being the demarcation necessitating immediate intervention | 10.1016/j.ijnss.2023.06.020 |
| 03 | Kayambankadzanja et al. | 2022 | Concept analysis | The proposed definition for critical illness is “Critical Illness is a state of ill health with vital organ dysfunction, a high risk of imminent death if care is not provided and the potential for reversibility | 10.1136/bmjopen-2022-060972 |
| 04 | Liang et al. | 2020 | Retrospective cohort study | Among patients with Covid-19 admitted to the hospital, critical illness was defined as the composite measure of admission to the intensive care unit, invasive ventilation, or death | 10.1001/jamainternmed.2020.2033 |
| 05 | Ostermann & Vincent | 2023 | Narrative Review | 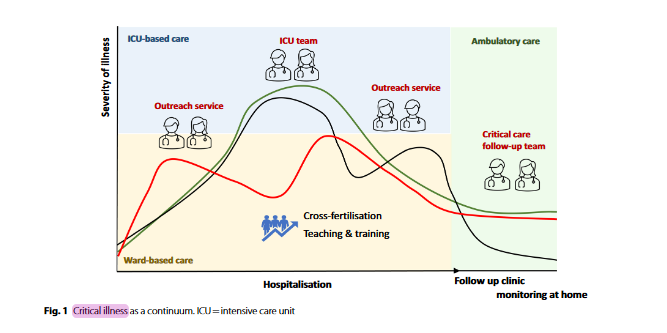Critical illness needs to be seen as a continuous and dynamic sequence of interlinked events from the very early moments of illness, through the stay in hospital, including ICU, and into recovery and rehabilitation. | 10.1186/s13054-023-04463-0 |
| 06 | Schweizerische Gesellschaft für Intensivmedizin | 2022 | Definition of a society | Ein Mensch ist kritisch krank, wenn sein Leben bedroht ist. Das kann daran liegen, dass ein oder gleichzeitig mehrere lebensnotwendige Organe wie etwa das Gehirn, das Herz oder die Lunge versagen. Doch auch eine akute schwere allgemeine Erkrankung wie eine Sepsis kann zur Folge haben, dass Organe innert kurzer Zeit nicht mehr richtig funktionieren oder unter Umständen sogar ausfallen. Aber auch eine akute Verschlechterung einer chronischen Erkrankung, ein Unfall oder ein medizinischer Eingriff können dazu führen, dass Organe nicht mehr so arbeiten, wie sie eigentlich sollen. Je grösser die Gefährdung, desto dringender wird die Behandlung in einem hochspezialisierten medizinischen Umfeld wie der Intensivstation mit ihrer modernen und effektiven Infrastruktur. Nur sie ermöglicht es, kritisch kranke Menschen rund um die Uhr zu überwachen und sofort auf deren gesundheitliche und psychische Bedürfnisse zu reagieren.  Je nach Schweregrad erfolgt die Behandlung der Betroffenen auf der Überwachungsstation, auch Intermediate Care Unit (IMC) genannt, oder – sollten mehrere Organe gleichzeitig betroffen sein – auf der mit mehr Behandlungsmöglichkeiten ausgestatteten Intensivstation | <https://www.sgi-ssmi.ch/de/patientinnenpatientenaufderIntensivstation.html> |
| 07 | Sharma et al. | 2019 | Narrative Review | Critical illness is any disease state, medical or surgical, that requires treatment in the intensive care unit. Although critical illness is frequently associated with infection or sepsis, other conditions such as severe trauma, the postsurgical state, pancreatitis, burn injury, hemorrhage, and ischemia can produce the same clinical findings as microbial invasion, even in the absence of an infectious organism. | 10.1002/ncp.10232 |
| 08 | Vincent | 2019 | Narrative Review | Critical illness is a dynamic continuum with various phases and trajectories, including deterioration, organ dysfunction and recovery or death.  Until relatively recently, critical illness was considered as a separate entity and the ICU, often a little cut-off from other areas of the hospital, was in many cases used as a last resort for patients so severely ill that it was no longer possible to care for them on the general ward. However, we are increasingly realizing that critical illness should be seen as just one part of the patient´s disease trajectory and how the patient is managed before and after ICU admission has an important role to play in optimizing outcomes. Identifying critical illness early before it reaches a stage where it is life-threatening, is a challenge and requires a combination of improved and more frequent or continuous monitoring of at-risk-patients, staff training to recognize when a patient is deteriorating, a system to call for help, and an effective response to that call. | 10.1186/s13054-019-2393-x |

**Table 3: Definitions of chronic critical illness**

| **Nr.** | **Author** | **Year** | **Study design** | **Definition** | **DOI/ Link** |
| --- | --- | --- | --- | --- | --- |
| 01 | Chadda & Puthucheary | 2024 | Review | - Post-intensive care syndrome, chronic critical illness, persistent critical illness and persistent inflammation, immunosuppression, and catabolism syndrome (PICS) are syndromes with overlapping features that require more precise definitions for diagnosis and study. - PICS is likely an endotype of chronic critical illness, and other endotypes likely exist.   - Post-intensive care syndrome   - Chronic critical illness   - Persistent critical illness   - Persistent inflammation, immunosupression and catabolism syndrome | https://doi.org/10.1016/j.bja.2023.11.052 |
| 02 | Demirkiran | 2021 | Retrospective cohort study | - Pediatric patients: ICU LOS ≥ 14 days and having at least one additional criterion - prolonged mechanical ventilation - tracheostomy - sepsis - severe wound (burn) or trauma - encephalopathy - traumatic brain injury - status epilepticus - being postoperative - neuromuscular disease - newborn ICU LOS ≥ 30 days in addition to prematurity | 10.1371/journal.pone.0248883. |
| 03 | Iwashyna et al. | 2015 |  | - At least five concepts:   - persistent critical illness (as defined here)   - CCI or medically complex patients (see below)   - diseases with long intrinsic recovery times   - prolonged weaning   - prolonged ICU length of stay (LOS). - Conceptual definition: patients whose reason for being in the ICU is now more related to their ongoing critical illness than their original reason for admission to the ICU. | https://doi.org/10.1016/S1441-2772(23)01492-8 |
| 04 | Kahn et al. | 2015 |  | - … defined CCI as an ICU length of stay of at least 8 days combined with at least one eligible diagnosis during the hospitalization.   - prolonged acute mechanical ventilation   - tracheotomy   - sepsis and other severe infections   - severe wounds   - multiple organ failure, ischemic stroke, intercerebral hemorrhage, or traumatic brain injury. | https://doi.org/10.1097/CCM.0000000000000710 |
| 05 | Loss et al. | 2017 | Review | New definitions have been proposed as follows   - - CCI   - persistent critical illness (PCI)   - diseases that necessarily require long recovery periods   - prolonged weaning from MV   - long ICU stay   Table 1 was extracted and modified from a 2010 review of diagnostic criteria for this syndrome  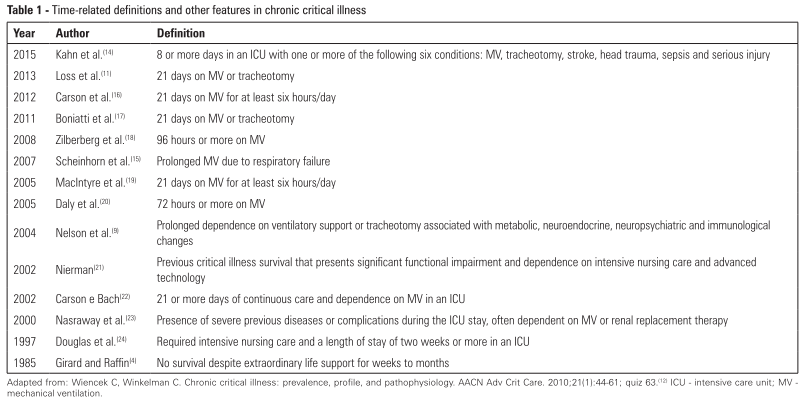 | https://doi.org/10.5935/0103-507X.20170013 |
| 06 | Marchioni et al. | 2020 | Observational prospective cohort study | Consensus definition resulted from the combination of two factors:   - ICU LOS ≥ 8 days, - and at least one out of five eligible conditions (MV prolonged >96 h; tracheostomy; sepsis or other severe infections; severe wounds and/or multiple organ failure; ischemic stroke, intercerebral hemorrhage or traumatic brain injury), (Carson, 2012) | https://doi.org/10.1016/j.pulmoe.2019.09.006 |
| 07 | Minton, Batten & Best | 2021 | Review | Seven definitions were identified   1. prolonged mechanical ventilation 2. failure to wean 3. insertion of tracheostomy 4. chronically critically ill 5. persistent critical illness 6. persistent inflammatory immunosuppressive and catabolic syndrome 7. frailty. | https://doi.org/10.1111/jocn.16078 |
| 08 | Morris et al. | 2024 | Review | ICU LOS of 10 days was identified as a surrogate marker for CCI. | https://doi.org/10.1016/j.amjsurg.2024.05.008 |
| 09 | Murphy Salem & Graham | 2021 | Review | A history of a prolonged PICU stay, ongoing acute care needs, and dependence on technology, or persistent multiorgan dysfunction (Definition according to Shapiro, Henderson, Hutton & Boss, 2017 | 10.3389/fped.2021.686206 |
| 10 | Shaw et al. | 2020 | Retrospective, Population-Based, Observational Study | Persistent Critical Illness has been defined as the point during an ICU stay when patients’ acute diagnoses and physiologic disturbance on ICU presentation are no longer more accurate at discriminating who will live from who will die than are patients’ baseline demographics and comorbidity. | https://doi.org/10.1097/cce.0000000000000102 |
| 11 | Zhang et al. | 2020 | Retrospective cohort study | Persistent critical illness was defined at the time when acute physiological characteristics were no longer more predictive of in-hospital mortality (i.e., vital status at hospital discharge) than antecedent characteristics. | 10.1186/s13054-020-2768-z |
| 12 | Zorko et al. | 2023 | Scoping Review | Definition for CCI that included concepts of PICU length of stay, medical complexity or chronic conditions, recurrent admissions, technology dependence, and uncertain prognosis | 10.1097/PCC.0000000000003125 |
